# β-catenin is overexpressed in hepatic fibrosis and blockage of Wnt/β-catenin signaling inhibits hepatic stellate cell activation

**DOI:** 10.3892/mmr.2014.2099

**Published:** 2014-04-01

**Authors:** WEN-SONG GE, YAO-JUN WANG, JIAN-XIN WU, JIAN-GAO FAN, YING-WEI CHEN, LIANG ZHU

**Affiliations:** 1 Department of Gastroenterology, Shanghai Xinhua Hospital, Shanghai 200092, P.R. China; 2 Shanghai Key Laboratory of Pediatric Gastroenterology and Nutrition, Shanghai 200092, P.R. China; 3 Shanghai Institute of Pediatric Research, Shanghai Jiao Tong University School of Medicine, Shanghai 200092, P.R. China; 4 Department of Gastroenterology, General Hospital of Jinan Military Command, Jinan, Shandong 250031, P.R. China; 5 Department of Gastroenterology, Shanghai Changzheng Hospital, Second Military Medical University, Shanghai 200003, P.R. China

**Keywords:** hepatic fibrosis, β-catenin, hepatic stellate cells, RNA interference

## Abstract

β-catenin, a core component of Wnt/β-catenin signaling, has been shown to be an important regulator of cellular proliferation and differentiation. Abnormal activation of Wnt/β-catenin signaling promotes tissue fibrogenesis. In the present study, the role of β-catenin during liver fibrogenesis was analyzed and the functional effects of β-catenin gene silencing in hepatic stellate cells (HSCs) using small interfering (si)RNA were investigated. The expression of β-catenin in human hepatic fibrosis tissues of different grades and normal human hepatic tissues was examined using immunohistochemistry. To inhibit the Wnt/β-catenin signaling pathway, siRNA for β-catenin was developed and transiently transfected into HSC-T6 cells using Lipofectamine 2000. β-catenin expression was evaluated by quantitative polymerase chain reaction (qPCR) and western blot analysis. The expression of collagen types I and III was evaluated by qPCR and immunofluorescent staining. Cellular proliferation and the cell cycle were analyzed using a methyl thiazolyl tetrazolium assay. Apoptosis was assessed by Annexin V staining. A higher expression level of β-catenin was identified in the patients with high-grade hepatic fibrosis in comparison with that of the normal controls. Additionally, β-catenin siRNA molecules were successfully transfected into HSCs and induced inhibition of β-catenin expression in a time-dependent manner. β-catenin siRNA treatment also inhibited synthesis of collagen types I and I in transfected HSCs. Furthermore, compared with those of the control group, siRNA-mediated knockdown of β-catenin in HSC-T6 cells inhibited cell proliferation and resulted in cell apoptosis. This study suggests a significant functional role for β-catenin in the development of liver fibrosis and demonstrates that downregulation of the Wnt/β-catenin signaling pathway inhibits HSC activation. Thus, this study provides a novel strategy for the treatment of hepatic fibrosis.

## Introduction

Hepatic fibrosis is an outcome of numerous chronic liver diseases, including hepatitis B and hepatitis C viral infections, alcoholic liver disease and non-alcoholic steatohepatitis (1). Hepatic fibrosis is a considerable medical problem associated with significant levels of morbidity and mortality (2). Regardless of the underlying etiology, hepatic fibrosis is characterized by the accumulation of excess extracellular matrix (ECM), including types I and III collagen. The extent of matrix deposition depends on the balance between the synthesis and degradation of ECM; when the levels of ECM synthesis exceed those of degradation, the pathological accumulation of ECM leads to liver fibrosis. The excessive ECM in the liver is mainly synthesized by activated hepatic stellate cells (HSCs) (3). Therefore, suppressing the activation of HSCs is essential for alleviation of liver fibrosis.

The Wnt/β-catenin signaling pathway is critical in development and in adult tissue homeostasis. Wnt/β-catenin signaling initiates a signaling cascade critical in the normal development of a number of organs, including the brain, intestines, skin, liver and lung (4). The hallmark of this pathway is that it activates the transcriptional role of the multifunctional protein β-catenin. Canonical Wnt signaling inactivates glycogen synthase kinase (GSK)-3β, preventing β-catenin phosphorylation. This action leads to accumulation of hypophosphorylated β-catenin in the cytoplasm, which subsequently translocates to the nucleus where it regulates target gene expression through interactions with members of the T-cell factor (TCF)/lymphoid enhancer factor (LEF) family of transcription factors (5). Previous studies have implicated Wnt/β-catenin signaling in abnormal wound repair and fibrogenesis. In addition, the Wnt canonical signaling pathway, mediated by β-catenin, has been indicated to be important in liver development and remodelling, and HSC activation (6–7). However, certain pathological processes, including fibrosis and liver malignancy, may occur partly due to the activation of the Wnt canonical pathway.

RNA interference is a powerful tool for post-transcriptional gene silencing (9) and has opened novel avenues in gene therapy. In the present study, the expression of β-catenin in hepatic fibrosis was detected in cases of hepatic fibrosis of different grades and normal hepatic tissues to confirm and explore the association between β-catenin and the progression of hepatic fibrosis. Furthermore, a synthetic small interfering RNA (siRNA) was transfected into HSC-T6 cells to suppress β-catenin expression and investigate whether inhibition of the Wnt/β-catenin signaling pathway attenuates hepatic fibrosis.

## Materials and methods

### Patients

All samples, from patients who underwent liver biopsies directed by ultrasonography within one week after inclusion in this study, were obtained from Shanghai Changzheng Hospital, Second Military Medical University (Shanghai, China) and Shanghai Xinhua Hospital, Shanghai Jiaotong University School of Medicine (Shanghai, China), between January 2006 and January 2007. The sample donation and application procedures were approved by the Health Human Research Ethics Committee of Shanghai Changzheng Hospital and the Health Human Research Ethics Committee of Xinhua Hospital. The study complied with the Declaration of Helsinki, 1995. The patients provided full written informed consent at the time of sample acquisition and patient anonymity has been preserved. All samples were treated by 10% formalin fixation and paraffin imbedding. Diagnosis and classification of the hepatic fibrosis were determined using Scheuer’s classification (10,11): F0, no fibrosis; F1, enlarged fibrotic portal tracts; F2, periportal or portal-portal septa, but intact architecture; F3, fibrosis with architectural distortion, but no obvious cirrhosis; F4, cirrhosis. All sections were blindly and independently assessed by three pathologists and the observed results were processed using the Kappa concordance test. The inter- and intraobserver results agreed excellently. A total of 89 samples were included: 15 normal cases of F0, 18 cases of F1, 20 cases of F2, 20 cases of F3 and 16 cases of F4.

### Histological and immunohistochemical examination

Part of all the paraffin-embedded liver tissues was stained with hematoxylin and eosin and another part underwent silver staining for reticular fibers. Immunohistochemical examination was conducted to detect the expression of β-catenin. Briefly, the paraffin sections of the left median hepatic lobes were incubated with 3% H_2_O_2_ in methanol at 37°C for 10 min to suppress endogenous peroxidase activity. Subsequent to blocking at room temperature for 20 min with 10% bovine serum (Wako, Osaka, Japan), the sections were incubated overnight at 4°C with antibodies against β-catenin (Santa Cruz Biotechnology, Inc., Santa Cruz, CA, USA), followed by incubation with horseradish peroxidase (HRP)-conjugated secondary antibody (Daco, Kyoto, Japan) at 37°C for 20 min. The signal was amplified by avidin-biotin complex formation and developed using diaminobenzidine followed by counterstaining with hematoxylin. The samples were dehydrated in alcohol and xylene and mounted onto glass slides. Positive cells were counted in at least 10 fields at ×400 magnification. The incidence of immunoreactivity for β-catenin was evaluated based on the mean percentage of cells positive, as follows: High, >80% of cells positive; intermediate, 25–80% of cells positive; low, <25% of cells positive and negative, <5% cells positive.

### Cell culture

HSC-T6, an immortalized rat HSC line exhibiting a stable phenotype and biochemical characteristics of activated HSCs, was donated by Dr SL Friedman (Liver Center Laboratory, San Francisco General Hospital, CA, USA) (12). All cells were maintained in RPMI-1640 medium with 10% fetal bovine serum in a humidified atmosphere at 37°C and 5% CO_2_. The cells were divided into four groups: A normal group (NG), in which cells were maintained in RPMI-1640 medium without transfection; a control group (CG), in which cells were transfected with non-specific gene silencing effects; and two treatment groups: in which cells were transfected with β-catenin siRNA and analyzed after either 24 or 48 h, respectively.

### β-catenin siRNA preparation and transfection

β-catenin-specific siRNAs were designed as described by Elbashir *et al* (13). The sense and antisense sequences of β-catenin siRNA were as follows: 5′-AAACTACTGTGG ACCACAAGCCCTGTCTC-3′ and 5′-AAGCTTGTGGTC CACAGTAGTCCTGTCTC-3′, respectively. The siRNA fragments were synthesized using the Silencer^®^ siRNA Construction kit (Ambion, Austin, Texas, USA) according to the manufacturer’s instructions. The cells were transfected with a mixture of plasmid DNA and Lipofectamine 2000 (Invitrogen Life Technologies, Carlsbad, CA, USA) in Opti-MEM I medium without serum (Invitrogen Life Technologies), as recommended by the manufacturer.

### Quantitative polymerase chain reaction (qPCR)

Total RNA was extracted at different time points following siRNA transfection using a TRIzol kit (Gibco Life Technologies) according to the manufacturer’s instructions. The mixture of RNA and primers was loaded into the PCR amplifier (PE5700; Perkin-Elmer, Norwalk, CT, USA). The following sense and antisense primers were used: Collagen I, 5′-GGTGGTTATGACTTCAGCTTCC-3′ and 5′-CATGTA GGCTACGCTGTTCTTG-3′; collagen III, 5′-GTCTTATCA GCCCTGATGGTTC-3′ and 5′-GCTCCATTCACCAGT GTGTTTA-3′; and β-actin, 5′-TGAAGGTCGGAGTCAACG GATTTGG-3′ and 5′-CATGTGGGCCATGAGGTCCAC CAC-3′. The PCR procedure was as follows: Predenaturate setting at 95°C for 5 min, denature at 94°C for 45 sec, annealing at 50 °C for 1 min and extension at 72°C for 1 min. The PCR was performed for 40 cycles followed by a final extension at 72°C for 10 min. The PCR product was then visualized by running it on a 1.5% agarose gel and was quantitatively analyzed with LabWorks 4.5 analysis software (UVP Products, Upland, CA, USA).

### Western blot analysis

Following transfection, the cells were harvested and immediately prepared for protein extraction. The protein content in the supernatant was detected using the bicinchoninic acid method (Pierce, Rockford, IL, USA). Equal quantities of protein were run on 10% SDS-PAGE gel and transferred to polyvinylidene fluoride membranes. Following incubation with 10% non-fat milk for 1 h, the membranes were probed with polyclonal rabbit anti-rat β-catenin antibody (1:400; Sigma, St. Louis, MO, USA) overnight at 4°C and then incubated with HRP-labeled goat anti-rabbit secondary antibodies (diluted 1:3,000; Santa Cruz Biotechnology, Inc.). The protein levels were normalized using β-actin as a loading control. The relative optical density of the protein bands was measured using a Zeineh Laser Densitometer (Biomed Instruments Inc., Fullerton, CA, USA) after subtracting the film background.

### Immunofluorescent staining

Expression of collagen types I and III in HSC-T6 cells infected with β-catenin siRNAs was examined by immunocytofluorescent staining using polyclonal antibodies against collagen types I and III (Boster Biological Tech Ltd., Fremont, CA, USA). The fixed cells were treated with the primary antibodies (against collagen types I and III) overnight at 4°C, followed by incubation with secondary antibodies (TRITC AffiniPure Goat Anti-Rabbit IgG; EarthOx, LLC, San Francisco, CA, USA) at 4°C for 2 h. The cells were then stained for 30 min at room temperature with 4,6-diamidino-2-phenylindole. Following rinsing, the slides were viewed with a Zeiss LSM-510 Laser Scanning Confocal microscope (Carl Zeiss AG, Oberkochen, Germany). The fluorescence was quantified with semi-quantitative analysis by image scanning.

### Cell proliferation and cell cycle analysis

The effect of siRNA-mediated downregulation of β-catenin on HSC-T6 cell proliferation was determined by 3-(4,5-dimethylthiazol-2-yl)-2,5-diphenyl tetrazolium bromide (MTT) assay. The cell suspension was placed into 96-well plates at 1,000 cells per well with eight repeat wells and incubated for 1, 2, 3, 4 or 5 days after transfection. The cells were then incubated with 20 μl methyl thiazolyl tetrazolium for 4 h. Following centrifugation, 150 μl dimethylsulfoxide was added to the precipitate and the absorbance of the enzyme was measured at 490 nm with a microplate reader (MK3; Multiskan Co., Vantaa, Finland). For the cell cycle analysis, HSC-T6 cells infected with β-catenin siRNA were harvested and fixed with 70% ethanol at 20°C for 24 h. Subsequently, 1×10^5^ cells were prepared to analyze the cell cycle phases by flow cytometry (ZM Coulter Counter; Coulter Electronics Inc., Hialeah, FL, USA).

### Apoptosis assessment by Annexin V staining

To detect the cells in the early stages of apoptosis, the Annexin V-fluorescein isothiocyanate (FITC) Apoptosis Detection kit (BD Biosciences, San Diego, CA, USA) was used according to the manufacturer’s instructions. Briefly, following transfection for 48 h, HSC-T6 cells were harvested and stained with propidium iodide and Annexin V-FITC in 100 μl staining solution at room temperature for 15 min in the dark. Samples were then diluted with binding buffer and were analyzed by the FACScalibur flow cytometer (BD Immunocytometry Systems, San Jose, CA, USA) within 1 h.

### Statistical analysis

Continuous data were expressed as the mean ± standard deviation. For statistical analysis, the group distributions were compared parametrically using Student’s t-test or one-way analysis of variance, and the group distributions were compared non-parametrically using the Mann-Whitney U test. The results were analyzed statistically using the Kruskal-Wallis test for the association between the incidence of immunoreactivity for β-catenin and the histological grade. P<0.05 was considered to indicate a statistically significant difference.

## Results

### Histological and immunohistochemical assessment of the hepatic fibrosis tissues

**Figure 1:**
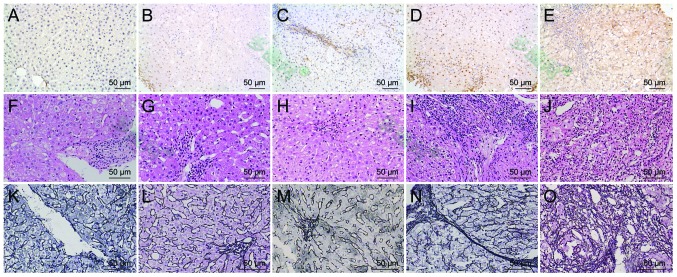
β-catenin was upregulated in liver fibrogenesis. (A–E) Immunohistochemical analysis of β-catenin distribution and expression in liver fibrosis specimens (original magnification, ×400). Brown color signifies positive expression. (A) No immunoreactivity was detected in the normal liver tissue; (B) weak staining in liver fibrosis tissue at F1; (C and D) moderate staining in liver fibrosis tissue at F2 and F3; (E) strong staining in liver fibrosis tissue at F4. (F–J) Hematoxylin and eosin was used to examine pathological alterations and collagen deposition. Images F–J indicate fibrosis stages F0, F1, F2, F3 and F4, respectively. (K–O) Silver staining of reticular fibers was used to examine pathological alterations and collagen deposition. Images K–O indicate fibrosis stages F0, F1, F2, F3 and F4, respectively. F0, no fibrosis; F1, enlarged fibrotic portal tracts; F2, periportal or portal-portal septa, but intact architecture; F3, fibrosis with architectural distortion, but no obvious cirrhosis; F4, cirrhosis.

**Figure 2:**
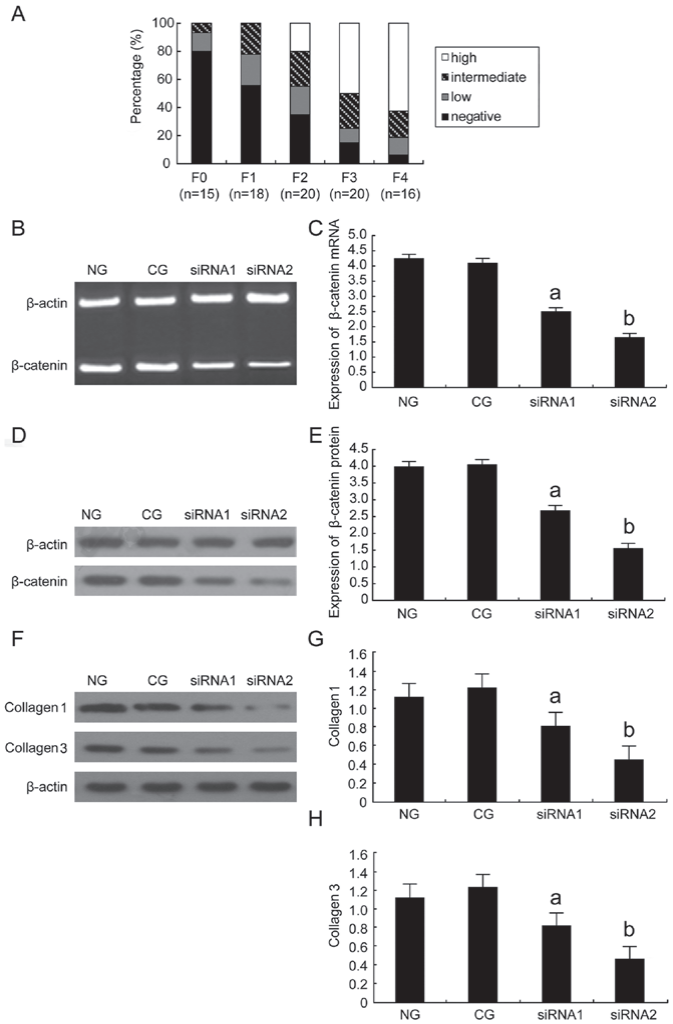
(A) Expression levels of β-catenin in different grades of hepatic fibrosis. F0, no fibrosis; F1, enlarged fibrotic portal tracts; F2, periportal or portal-portal septa, but intact architecture; F3, fibrosis with architectural distortion, but no obvious cirrhosis; F4, cirrhosis. Incidence of immunoreactivity for β-catenin: High, >80% of cells positive; intermediate, 25–80% of cells positive; low, <25% of cells positive and negative, <5% cells positive. (B–E) β-catenin siRNA inhibited β-catenin mRNA and protein expression in HSC-T6 cells compared with NG and CG cells. β-actin served as the internal loading control. (B) qPCR analysis for β-catenin mRNA expression in HSC-T6 cells following β-catenin siRNA transfection. (C) Semi-quantitative analysis of the qPCR result. (D) Western blot analysis of β-catenin protein expression following transfection. (E) Semi-quantitative analysis of the western blotting results. (F–H) β-catenin siRNA inhibited collagen types I and III mRNA expression in HSC-T6 cells. (F) qPCR analysis for collagen types I and III mRNA expression in HSC-T6 cells following siRNA β-catenin transfection. β-actin served as the internal loading control. (G and H) Semi-quantitative analysis of the qPCR result. ^a^P<0.05, ^b^P<0.01 compared with NG and CG. n, number of samples; siRNA, small interfering RNA; NG, normal group; CG, control group; qPCR, quantitative polymerase chain reaction. siRNA1, 24 h after transfection; siRNA2, 48 h after transfection.

Histologically, the hepatic fibrosis tissues exhibited connective tissue fibers extending from the central vein, and early septal formation was established in the livers of the F2 group. The expression levels of β-catenin were examined in the tissues by immunohistochemistry. The majority of β-catenin staining was observed in the high grade hepatic fibrosis tissues. Little specific β-catenin staining was observed in normal liver tissues (Fig. 1). Compared with that of the normal controls, β-catenin was overexpressed in hepatic fibrosis and its expression level increased with the histological grade of the fibrosis (P<0.05; Fig. 2A). The data suggested that overexpression of β-catenin may be associated with the degree of liver fibrosis and may be important in the initiation and progression of hepatic fibrosis.

### β-catenin siRNA effectively inhibits β-catenin expression in HSC-T6 cells

Forty-eight hours after transfection with β-catenin siRNA, the β-catenin transcript and protein levels were reduced in the transfected cells compared with those in the control group. This β-catenin gene-silencing effect was reproducible and specific as it failed to knock down the expression of an unrelated protein, β-actin. The β-catenin siRNA used in the present study was able to reduce β-catenin mRNA expression levels in HSC-T6 cells compared with the levels in the NG and CG (Fig. 2B). Western blotting further confirmed the β-catenin siRNA silencing of the β-catenin protein in the HSC-T6 cells (Fig. 2D). Semi-quantitative analysis of the qPCR and western blotting results (Fig. 2C and E) also revealed that β-catenin siRNA reduced the expression levels of β-catenin in HSC-T6 cells comapared with those in the NG or CG.

### β-catenin siRNA downregulates collagen types I and III in HSC-T6 cells

**Figure 3:**
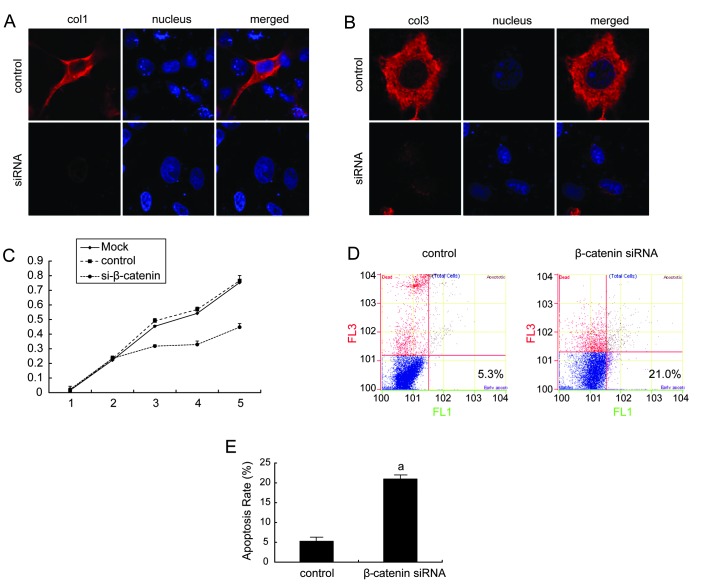
(A and B) β-catenin siRNA inhibited collagen types I and III mRNA expression in HSC-T6 cells. Immunofluorescence staining analysis for collagen (A) type I and (B) type III expression in HSC-T6 cells following siRNA β-catenin transfection. (C) β-catenin siRNA suppressed HSC-T6 cell proliferation. Cell growth curves of HSC-T6 cells transfected with β-catenin siRNA were analyzed by MTT conversion. Each sample was analyzed in triplicate and error bars are included. Compared with that of the control and non-transfection groups, the proliferation of the β-catenin siRNA group was significantly reduced at 3, 4 and 5 days after transfection (P<0.05). (D and E) Annexin V analysis of apoptosis induced by β-catenin siRNA in HSC-T6 cells. (D) Example of the apoptotic effect of β-catenin siRNA in HSC-T6 cells. (E) The percentage of apoptotic cells was increased following treatment with β-catenin siRNA compared with that of the control group. ^a^P<0.01, compared with control siRNA group. Error bars indicate standard deviation. siRNA, small interfering RNA; MTT, 3-(4,5-dimethylthiazol-2-yl)-2,5-diphenyl tetrazolium bromide.

To investigate the effect of β-catenin siRNA on collagen degradation, the quantity of collagen types I and III in HSC-T6 cells transfected with β-catenin siRNA was examined using qPCR and immunofluorescent staining. The expression levels of collagen types I and III mRNAs were reduced in the HSC-T6 cells transfected with β-catenin siRNA, compared with those in the cells of the NG or CG (Fig. 2F–H). The fluorescent light for collagen types I and III observed in the cell cytoplasm was significantly reduced in the β-catenin siRNA group compared with that in the control group. The expression of collagen type I was reduced by 82% and the expression of collagen type III was reduced by 78% by β-catenin siRNA treatment using quantification of the fluorescent light intensity compared with that in the controls (Fig. 3A–B).

### β-catenin siRNA inhibits the proliferation of HSC-T6 cells

To investigate whether the reduction in β-catenin levels exhibits an effect on cell growth and proliferation, cell cycle distribution and MTT assays were performed. Knockdown of β-catenin by siRNA at 3, 4 and 5 days after transfection led to reduced cell proliferation in the treatment group than that in the control and mock groups (Fig. 3C). Investigation of the cell cycle also indicated that cells were arrested in the G0/G1 phase and the proportion of cells in the S phase was significantly reduced compared with the control group following downregulation of β-catenin in the HSCs (Table I).

### β-catenin siRNA induces apoptosis in HSC-T6 cells

Annexin V staining revealed that the proportion of apoptotic HSC-T6 cells transfected with β-catenin siRNA was significantly elevated when compared with the proportion of apoptotic cells in the control group (Fig. 3D–E). The rate of apoptosis increased from 5.3% in the control group to 21.0% in the β-catenin siRNA group (P<0.01). This result suggested that siRNA targeting of β-catenin was able to induce apoptosis in HSC-T6 cells.

## Discussion

Hepatic fibrosis is a chronic pathological process that involves an inflammatory response characterized by an excessive release of inflammatory cytokines and infiltration of inflammatory cells (14,15). All have been demonstrated to promote the activation of HSCs and deposition of ECM in the liver, leading to liver fibrosis (16). HSCs are important in ECM remodeling and have been used as an *in vitro* cell model of hepatic fibrosis for over a decade (12). HSCs are the most important source of ECM proteins during this fibrotic process. Therefore, the majority of antifibrotic therapies are designed to inhibit the activation, proliferation or synthetic products of HSCs.

The Wnt signaling pathway is an evolutionarily conserved, complex signaling pathway that is critical for development, differentiation and cellular homeostasis (17). Three signal transduction pathways are activated by Wnt: The canonical Wnt pathway, the planar cell polarity pathway and the Wnt/Ca^2+^ pathway (18). The canonical Wnt/β-catenin pathway is centered on regulating the levels of its predominant effector, β-catenin (19). In the absence of Wnt ligands, β-catenin is sequestered in the cytoplasm by a protein complex composed of the scaffolding protein Axin, adenomatous polyposis coli, casein kinase 1 (CK1) and GSK3β. Specific serine and threonine residues in the amino terminal region of β-catenin are sequentially phosphorylated by CK1 and GSK3β. Phosphorylated β-catenin is then ubiquinated and targeted for proteasomal degradation (20). As a result of this continuous degradation, β-catenin is prevented from accumulating in the cytoplasm and therefore from reaching the nucleus. In the absence of nuclear β-catenin, Wnt-target genes are repressed by binding of the TCF/LEF family of proteins. Activation of the canonical pathway results in inhibition of GSK3 and allows stabilization of cytosolic β-catenin and its translocation to the nucleus, where it binds to the TCF/LEF family of transcription factors to stimulate the expression of multiple Wnt target genes, including c-myc, c-jun and cyclin D1 (18,21). Wnt signals are also regulated by secreted antagonists that either bind directly to the ligand, including Wnt inhibitory factor and the secreted frizzled (Fz)-related protein family, or prevent lipoprotein receptor-related protein (LRP) coreceptor association with Fz. Dickkopf (Dkk), the best-characterized of the latter group of antagonists, binds to LRP6 and inhibits the Wnt-induced Fz-LRP5/6 complex formation that is essential to the canonical pathway. Canonical Wnt signaling through β-catenin is facilitated by Wnt family members, including Wnt1, Wnt3a and Wnt10b and Fz receptors, such as Fz1 and Fz5 (21,22).

Wnt/β-catenin is an evolutionarily conserved cellular signaling system essential in the pathogenesis of numerous human diseases (5,23). Wnt signaling has been implicated in human fibrotic diseases, including pulmonary and renal fibrosis (4,24). Since Wnt activity is enhanced in liver cirrhosis (25), it is also likely that Wnt signaling is involved in hepatic fibrogenesis. The importance of the Wnt/β-catenin pathway in the liver only began to be recognized in the late 1990’s (26). As in other organs, the Wnt/β-catenin pathway is critical for liver development. Certain studies have suggested that the pathway is also important in liver regeneration, liver metabolism and maintenance of normal function in the adult liver (27–29). Aberrant activation of β-catenin has been implicated in the pathogenesis of hepatobiliary neoplasias, ranging from benign lesions to liver cancer (8,30–33). One study used DNA microarrays to investigate gene-expression differences between quiescent and activated rat HSCs; the levels of the ligands Wnt4 and Wnt5 and the Wnt receptor Fz-2 were found to be upregulated (6). In another study, increased expression levels of canonical Wnt3a and 10b and noncanonical (Wnt4 and Wnt5a) Wnt genes, along with Fz-1 and -2 as well as Fz coreceptors, were identified in activated HSCs, but not in quiescent HSCs (34). However, higher levels of nuclear β-catenin and TCF DNA binding were found in activated HSCs, suggesting that canonical Wnt signaling is important in HSC activation. Since treatment with the inhibitors of Wnt signaling, Dkk-1 and Chibby, was able to restore quiescence of HSCs in culture, and high expression levels of Dkk-1 induced apoptosis of cultured HSCs, these results raise the possibility that inhibition of Wnt signaling may be a potential therapeutic strategy for preventing liver fibrosis.

Inhibition of the Wnt pathway has also been analyzed in the regulation of fibrotic responses in tissues. When Dkk, the best characterized naturally secreted antagonist of the Wnt pathway, was administered locally in a naked plasmid vector for gene therapy, blockade of canonical Wnt pathway inhibited renal interstitial fibrosis (35). Inhibition of the Wnt/β-catenin pathway by β-catenin siRNA also reduced bleomycin-induced pulmonary fibrosis in mice (36). In the present study, β-catenin expression in hepatic fibrosis was verified and the correlation between β-catenin and hepatic fibrosis progression was explored. Following silencing of β-catenin in HSCs by siRNA, there was found to be a reduction in the levels of collagen types I and III mRNA and protein expression, demonstrating that inhibition of β-catenin directly results in suppression of HSC activation and collagen synthesis. β-catenin siRNA was also found in the regulation of cell proliferation and in the cell cycle. To the best of our knowledge, this is the first study that has used siRNA targeting of β-catenin to inhibit HSC activation. As β-catenin is multifunctional with multiple molecular interactions, the definitive mechanism responsible remains uncertain.

In conclusion, β-catenin was upregulated during liver fibrogenesis. A reduction in the β-catenin expression levels in HSCs with siRNA suppressed the activity of HSCs and collagen synthesis, and enhanced collagen degradation. The results of the present study suggest a significant functional role for β-catenin in the development of liver fibrosis and that targeted knockdown of β-catenin mRNA may have a therapeutic effect on hepatic fibrosis.

## Figures and Tables

**Table I:** Effect of β-catenin siRNA on the cell cycle (%).

Cell cycle phase	β-catenin siRNA group	Control group
G0/G1	59.42±0.46^a^	43.52±0.65
S	28.68±1.28^a^	42.56±1.62
G2/M	13.28±1.06	14.38±1.26

a P<0.05 compared with control group.

Values are presented as the mean ± standard deviation. siRNA, small interfering RNA.
